# Prevention of Pulmonary Complications of Pneumoperitoneum in Rats

**DOI:** 10.1186/1749-8090-6-14

**Published:** 2011-02-08

**Authors:** Sami Karapolat, Suat Gezer, Umran Yildirim, Talha Dumlu, Banu Karapolat, Ismet Ozaydin, Mehmet Yasar, Abdulkadir Iskender, Hayati Kandis, Ayhan Saritas

**Affiliations:** 1 Department of Thoracic Surgery, Duzce University School of Medicine, Duzce, Turkey; 2 Department of Pathology, Duzce University School of Medicine, Duzce, Turkey; 3 Department of Pulmonary Diseases, Duzce University School of Medicine, Duzce, Turkey; 4 Department of General Surgery, Duzce University School of Medicine, Duzce, Turkey; 5 Department of Anesthesiology and Reanimation, Duzce University School of Medicine, Duzce, Turkey; 6 Department of Emergency Medicine, Duzce University School of Medicine, Duzce, Turkey

## Abstract

**Background:**

Carbon dioxide (CO_2_) pneumoperitoneum facilitates the visualization of abdominal organs during laparoscopic surgery. However, the associated increase in intra-abdominal pressure causes oxidative stress, which contributes to tissue injury.

**Objective:**

We investigated the ability of the antioxidant and anti-inflammatory drug Erdosteine to prevent CO_2 _pneumoperitoneum-induced oxidative stress and inflammatory reactions in a rat model.

**Methods:**

Fourteen female adult Wistar albino rats were divided into a control group (Group A, *n *= 7) and an Erdosteine group (Group B, *n *= 7). Group A received 0.5 cc/day 0.9% NaCl, and Group B received 10 mg/kg/day Erdosteine was administered by gavage, and maintained for 7 days prior to the operation. During the surgical procedure, the rats were exposed to CO_2 _pneumoperitoneum with an intra-abdominal pressure of 15 mmHg for 30 min. The peritoneal gas was then desufflated. The rats were sacrificed following 3 h of insufflation. Their lungs were removed, histologically evaluated, and scored for intra-alveolar hemorrhage, alveolar edema, congestion, and leukocyte infiltration. The results were statistically analyzed. A value of *P *< 0.05 was considered statistically significant.

**Results:**

Significant differences were detected in intra-alveolar hemorrhage (*P *< 0.05), congestion (*P *< 0.001), and leukocyte infiltration (*P *< 0.001) in Group A compared with Group B. However, the differences in alveolar edema were not statistically significant (*P *= 0.698).

**Conclusions:**

CO_2 _pneumoperitoneum results in oxidative injury to lung tissue, and administration of Erdosteine reduces the severity of pathological changes. Therefore, Erdosteine may be a useful preventive and therapeutic agent for CO_2 _pneumoperitoneum-induced oxidative stress in laparoscopic surgery.

## Introduction

Laparoscopic surgical techniques have long been favored in many therapeutic and diagnostic procedures because they offer a range of advantages compared with conventional open techniques. These include less extensive trauma and discomfort to the patient, decreased duration of hospitalization, minimal wound problems, better cosmetic results, fewer postoperative pulmonary complications, and shorter time to recovery [1,2]. This minimally invasive procedure generally requires a pneumoperitoneum for adequate visualization and exposure of the structures to be operated upon. Many gases such as helium, argon, N_2_O, and CO_2 _have been used for the creation of pneumoperitoneum.

Currently, CO_2 _is usually used for insufflation due to its low cost, nonflammabity, chemical stability, and high diffusion capacity with subsequent rapid absorption and excretion [3]. CO_2 _is also highly soluble and, therefore, poses a lower risk of gas embolism. However, CO_2 _pneumoperitoneum also causes an increase in intra-abdominal pressure above the normal physiological portal circulation pressure (7-10 mmHg), resulting in splanchnic ischemia. During laparoscopy, there is a marked reduction in blood flow to the hepatic, renal, and intestinal circulatory systems. When the laparoscopic procedure is completed, abdominal deflation is performed. This reduces the intra-abdominal pressure and increases splanchnic perfusion. During reperfusion, free oxygen radicals, which are the most important mediators of oxidative tissue damage and consequential organ dysfunction, are generated as a result of ischemia-reperfusion induced by the inflation and deflation of the pneumoperitoneum [4]. In general, the most likely causes of oxidative stress as a consequence of CO_2 _pneumoperitoneum are ischemia-reperfusion injury due to changes in the abdominal pressure, inflammation associated with tissue trauma, and diaphragmatic dysfunction [5]. Oxidative stress damages cellular components, causing microvascular leakage and lipid peroxidation of cellular membranes. This in turn generates more free radicals, with a self-propagating cycle leading to pathological changes ranging from edema and cell injury to cell death by necrosis.

Finally, CO_2 _pneumoperitoneum can affect several homeostatic systems, leading to alterations in the acid-base balance, blood gases, hepatic perfusion, and cardiovascular and pulmonary physiology [6]. Frequently, hypercapnia, acidosis, and systemic and pulmonary hypertension occur. Organ dysfunction may also occur in splanchnic organs and even remote organs such as the lungs. As reported previously, pulmonary complications of CO_2 _pneumoperitoneum are represented by hypoxemia, barotrauma, pulmonary edema, and atelectasis [4]. These problems are well tolerated in most patients. Nevertheless, older patients and those with conditions such as emphysema and chronic obstructive pulmonary disease are at risk for depressed pulmonary function and an increased rate of perioperative complications. Thus, the reduction or prevention of CO_2 _pneumoperitoneum-induced oxidative stress and inflammatory reactions by antioxidant and anti-inflammatory drugs may be useful for these patients in clinical practice.

With these issues in mind, we administered prophylactic Erdosteine prior to CO_2 _pneumoperitoneum in rats. To our knowledge, this is the first study of this drug for the treatment of pulmonary complications of CO_2 _pneumoperitoneum.

## Methods

### Population

A prospective, randomized, double-blinded, controlled, experimental study was conducted with 14 female adult Wistar albino rats from the same colony weighting 220-250 g. The rats were obtained from the Experimental Animals Laboratory of Duzce University Faculty of Medicine. The purpose of using rats is easy availability, safety, and the high ratio of repeating the experiment.

### Design

The rats were randomly divided into two groups: Group A: control (*n *= 7) and Group B: Erdosteine (*n *= 7). They were maintained under specific pathogen-free conditions to avoid infections and housed separately in a light-controlled room with a 12:12 h light-dark cycle. The temperature (22 ± 0.5°C) and relative humidity (65-70%) were kept constant. Unnecessary stresses were avoided throughout the study. Standard laboratory rodent chow and water were available ad libitum. The animals had not been used in another study or been given any drugs previously. They were deprived of food for 12 h before the experiment but had free access to water.

Group A received 0.5 cc/day 0.9% NaCl, and Group B received 10 mg/kg/day Erdosteine (Erdostin, Sandoz, Turkey) was administered by gavage, and maintained for 7 days prior to the operation day. All of the rats were anesthetized by administering ketamine hydrochloride (Ketalar, Pfizer, Turkey) 50 mg/kg and xylazine hydrochloride (Rompun, Bayer, Turkey) 3 mg/kg intraperitoneally. During the procedure, additional doses were administered if necessary. The experiments were performed in a position allowing spontaneous breathing under sterile conditions. The body temperature was maintained at 37.0°C with a heat pad to prevent the effects of hypothermia and to maintain the stability of hemodynamic parameters.

During the procedure, the animals were placed in a supine position. A Veress needle was placed supraumbilically into the peritoneal cavity, and a pneumoperitoneum was established via the insufflation of CO_2 _by a CO_2 _insufflator. The intra-abdominal pressure was set at 15 mmHg. As a result of a decrease in the intra-abdominal pressure due to peritoneal CO_2 _absorption or CO_2 _leakage close to the needle, CO_2 _was automatically insufflated into the peritoneal cavity to maintain the intra-abdominal pressure at the desired level. The pneumoperitoneum was maintained for 30 minutes, and the peritoneal gas was then desufflated. The rats were sacrificed by intraperitoneal administration of lethal ketamine hydrochloride after 3 hours of insufflation.

The lungs of the rats were removed by median sternotomy. The specimens were promptly fixed in 10% formalin, dehydrated in graded concentrations of ethanol, cleared in xylene, and processed for paraffin embedding. At least six tissue sections 5 μm thick were obtained. Light microscopy was used for histopathological analysis of the Hematoxylin-Eosin stained sections. One blinded pathologist analyzed the samples.

Each lung tissue was evaluated for histopathological changes, including intra-alveolar hemorrhage, alveolar edema, congestion, and leukocyte infiltration. Intra-alveolar hemorrhage, alveolar edema, and congestion were scored on a scale from 0 to 3, where 0 = absence of pathology (<5% of maximum pathology), 1 = mild (<10% of maximum pathology), 2 = moderate (15-20% of maximum pathology), and 3 = severe (20-25% of maximum pathology) [7]. Leukocyte infiltration was evaluated to determine the severity of inflammation resulting from pneumoperitoneum. Each section was divided into 10 subsections, and leukocyte infiltration was examined in each of the subsections at a magnification of 400× with the following scale: 0, no extravascular leukocytes; 1, <10 leukocytes; 2, 10-45 leukocytes; 3, >45 leukocytes. An average of the numbers was used for comparison [7,8].

### Ethics

The study was approved by a local ethics board of Duzce University Faculty of Medicine, Animal Care and Use Committee in 2009. The rats were cared for in accordance with the *Guide for the Care and Use of Laboratory Animals*.

### Statistical analysis

The results were recorded by the principal investigator and analyzed statistically upon completion of the study. The statistical analysis was performed with SPSS software, version 11.5 (SPSS, Inc., Chicago, IL). Clinical data were expressed as the median ± the standard error of mean (minimum-maximum). The parametric Student's *t*-test was used for group comparison, and a *P *value less than 0.05 was considered statistically significant.

## Results

All 14 rats survived the time to the study start date and the surgical procedure. Macroscopic examination of the lungs following removal showed that all specimens were normal in both groups.

The specimens were histologically evaluated and scored for intra-alveolar hemorrhage, alveolar edema, congestion, and leukocyte infiltration. The scores of intra-alveolar hemorrhage, congestion, and leukocyte infiltration were lower in Group B than Group A. However, the scores of alveolar edema in both groups were similar. All of the scores are presented in Table 1.

**Table 1:** Histopathological scores of Group A and Group B

***Rat No***	***Intra-alveolar hemorrhage***	***Alveolar edema***	***Congestion***	***Leukocyte infiltration***
***Group A-1***	1	0	3	2
***Group A-2***	1	1	3	3
***Group A-3***	1	0	2	3
***Group A-4***	1	0	2	2
***Group A-5***	1	1	3	3
***Group A-6***	1	1	3	2
***Group A-7***	1	1	3	2
				
***Group B-1***	0	0	1	1
***Group B-2***	0	0	1	1
***Group B-3***	1	0	2	2
***Group B-4***	0	1	2	1
***Group B-5***	1	2	2	1
***Group B-6***	1	0	2	1
***Group B-7***	1	0	1	2

**Figure 1:**
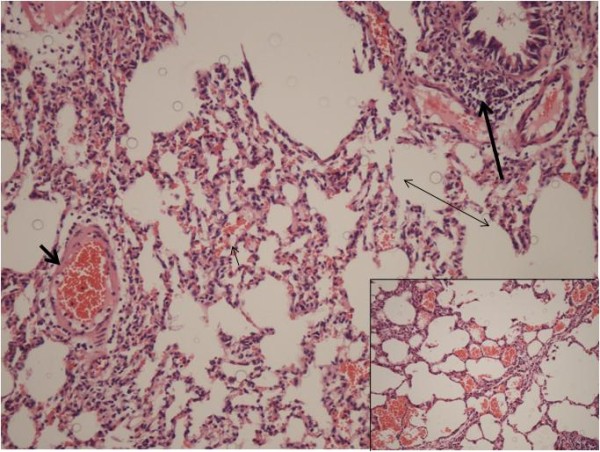
**Photomicrograph of histopathology from Group A (Control) displaying increased intra-alveolar hemorrhage (thin short arrow), congestion (thick short arrow), and leukocyte infiltration (thick long arrow)**. Alveolar edema (double arrow) was slight. (Hematoxylin-Eosin, original magnification × 20)

**Figure 2:**
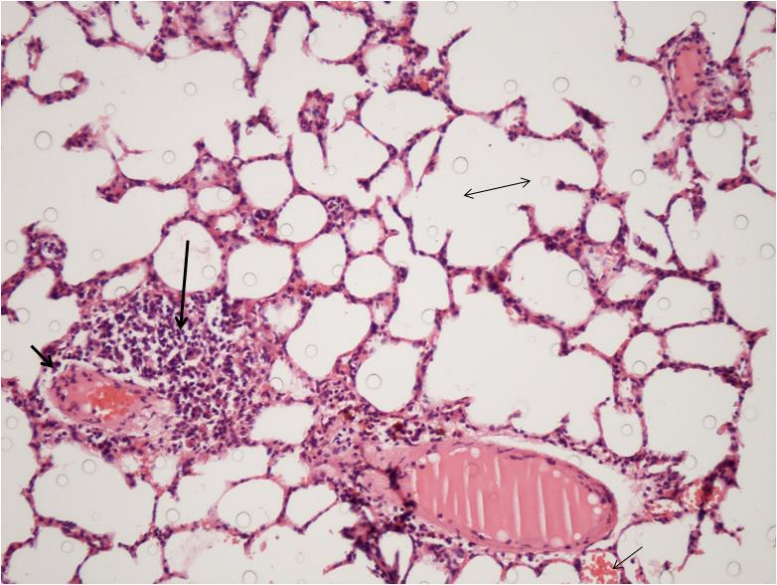
**Photomicrograph of histopathology from Group B (Erdosteine) displaying decreased intra-alveolar hemorrhage (thin short arrow), congestion (thick short arrow), and leukocyte infiltration (thick long arrow)**. Alveolar edema (double arrow) was slight. (Hematoxylin-Eosin, original magnification × 20)

Analysis of the specimens from Group A revealed diffuse intra-alveolar hemorrhage. In addition, dense congestion and leukocyte infiltration were present. Slight alveolar edema was detected around the congestion areas. Analysis of Group B specimens showed less intra-alveolar hemorrhage, congestion, and leukocyte infiltration, especially in alveolar subepithelial regions. Overall, alveolar edema in this group was almost the same as Group A. Histopathological photographs of the sections are shown in Figures 1 &2.

All of the histopathological results were statistically analyzed for significance. Significant differences were detected in intra-alveolar hemorrhage (*P *< 0.05), congestion (*P *< 0.001), and leukocyte infiltration (*P *< 0.001) in Group A compared with Group B, with the pathological changes reduced in the latter group. However, the differences in alveolar edema were not statistically significant (*P *= 0.698) (Table 2).

**Table 2:** Results of statistical analysis (Median ± SEM)

***Parameter***	***Group A Control (n = 7)***	***Group B Erdosteine (n = 7)***
***Intra-alveolar hemorrhage***	1.00 ± 0.00	0.57 ± 0.53
***Alveolar edema***	0.57 ± 0.53	0.43 ± 0.79
***Congestion***	2.71 ± 0.49	1.57 ± 0.53
***Leukocyte infiltration***	2.43 ± 0.53	1.29 ± 0.49

## Discussion

This study of an experimental CO_2 _pneumoperitoneum model revealed three points: (a) The predicted antioxidant and anti-inflammatory effects of Erdosteine were achieved, and histopathological analysis of intra-alveolar hemorrhage and congestion in the lungs revealed better results in Group B. (b) Leukocyte infiltration was reduced in Group B. (c) Erdosteine did not affect the intensity of alveolar edema in the lungs.

In general, CO_2 _pneumoperitoneum induces hemodynamic, pulmonary, renal, splanchnic, and endocrine pathophysiological changes. In some patients, complications can develop depending on intra-abdominal pressure, the amount of CO_2 _absorbed, the circulatory volume of the patient, the ventilation technique used, the underlying pathological conditions, and the type of anesthesia used [4]. During laparoscopy, an intra-abdominal pressure as high as 8-20 mmHg is produced and maintained. Increased intra-abdominal pressure as low as 10 mmHg causes a considerable decrease in splanchnic blood flow. The deflation of the pneumoperitoneum reduces the intra-abdominal pressure and increases splanchnic perfusion, yielding an ischemia-reperfusion model capable of generating free radicals during the early phase of reperfusion and causing reperfusion injury [9]. It is well known that ischemia causes considerable tissue damage, which is exacerbated by reperfusion with oxygenated blood [10].

This ischemia-reperfusion injury is not only limited to the organs experiencing ischemia-reperfusion but also involves distant organs that are not directly affected by ischemia-reperfusion. As a result of the migration of inflammatory cells such as macrophages, neutrophils, and lymphocytes, platelets, fibroblasts, and epithelial cells join forces to repair the injured tissue. However, the free oxygen radicals (H_2_O_2_, O_2_^-^, and OH^-^) and proteases released from the accumulated inflammatory cells, especially neutrophils can increase the systemic availability of inflammatory mediators, leading to leukocyte activation and endothelial adhesion molecule expression and vascular endothelial damage of remote organs. The free oxygen radicals are capable of reacting with proteins, nucleic acids, and lipids resulting in lipid peroxidation of biological membranes [11].

Various organs may control or prevent the damaging effects of oxidant species by enzymatic and nonenzymatic antioxidant defense. However, the antioxidant defenses of the human body are unable to combat fully the effects of oxidative stress. Therefore, cells contain systems that can repair deoxyribonucleic acid following attack by radicals, degrade proteins damaged by radicals, and metabolize lipid hydroperoxides in membranes [12]. Different strategies such as the establishment of low intra-abdominal pressure, insufflation with different gases, and drugs that support the body's auto defense mechanisms are useful to prevent CO_2 _pneumoperitoneum-induced oxidative stress and inflammatory reactions. Researchers have used various approaches to prevent this problem. In their experimental study, Yilmaz et al. compared the levels of free radical production and antioxidant status with a pneumoperitoneum based on helium and CO_2_, different values of intra-abdominal pressure. They found that CO_2 _pneumoperitoneum produced higher malondialdehyde and carbonyl responses and resulted in greater sulphydryl consumption and that helium limited the postoperative oxidative response following laparoscopy [13]. Uzunkoy et al. administered isothermic or hypothermic CO_2 _pneumoperitoneum to 30 elective laparoscopic cholecystectomy subjects and performed respiratory function tests in the preoperative period and at 12h following the operation. They concluded that pneumoperitoneum created with isothermic CO_2 _resulted in fewer negative effects and rapid postoperative improvement and suggested that isothermic CO_2 _pneumoperitoneum may be preferable in routine clinical practice for patients with respiratory problems [2]. Nesek-Adam et al. measured several biochemical parameters including liver enzymes to determine the effect of low-pressure pneumoperitoneum and pentoxifylline on oxidative stress in rabbits. They found that low-pressure pneumoperitoneum attenuates ischemia-reperfusion injury and that pretreatment with pentoxifylline does not prevent the development of oxidative stress [1]. In contrast to these findings, Dinckan et al. reported in their experimental study that pentoxifylline could reduce CO_2 _pneumoperitoneum-induced peritoneal oxidative stress [14]. In addition, Ypsilantis et al. previously demonstrated that prophylaxis with the antioxidant agent mesna prevented oxidative stress in the splanchnic organs of rats undergoing CO_2 _pneumoperitoneum treatment [10].

In our study, we aimed to prevent CO_2 _pneumoperitoneum-induced oxidative stress and inflammatory reactions by using Erdosteine, a multifactorial drug with antibacterial, anti-inflammatory, and antioxidant properties that can decrease inflammation and oxidative tissue damage, while taking the physiopathological process of CO_2 _pneumoperitoneum into consideration.

The popularity of Erdosteine is mainly associated with its mucolytic and mucokinetic properties. The drug contains two blocked sulfhydryl groups. Following hepatic metabolization to the active species called Metabolite 1 (Met 1) and opening of the thiolactone ring, one of the groups contributes to free radical scavenging and antioxidant effects [15,16]. Met 1 has been shown to inhibit nitric oxide, superoxide, and peroxynitrite production in vitro during respiratory burst of human neutrophils [15-17]. The main mechanism of action of Erdosteine may be related to its ability to inhibit some inflammatory mediators and some proinflammatory cytokines that are specifically involved in oxidative stress and in cell membrane damage [17]. Erdosteine prevents the accumulation of free oxygen radicals when their production is accelerated and increases antioxidant cellular protective mechanisms. In doing so, the drug protects tissues by reducing lipoperoxidation, elastase activity, neutrophil infiltration, and cell apoptosis [18,19]. The efficacy and tolerability of Erdosteine have been demonstrated over a number of years [19]. Patients may experience a low incidence of side effects, most of which are gastrointestinal and generally mild.

We initiated Erdosteine treatment 7 days before the operation day and maintained the treatment until the operation day. We selected this 7-day regime because previous studies have shown that Erdosteine when administered for 4 days resulted in a substantial decline in the concentration of both reactive oxygen species and cytokines in patients with stable chronic obstructive pulmonary disease. They have also demonstrated a significant reduction in the level of 8-isoprostane (a product of lipid peroxidation) following treatment for 7 days [20,21]. Several experimental studies have also shown that Erdosteine at 10 mg/kg/day provides sufficient efficacy [16,18].

Based on histological analyses, we found decreased levels of intra-alveolar hemorrhage in Group B. In general, CO_2 _pneumoperitoneum-induced oxidative stress caused damage to pulmonary tissue and alveolar epithelium cells, as well as endothelial arteriole and venule cells, leading to intra-alveolar hemorrhage with disruption of alveoli. The severity of such intra-alveolar hemorrhage is directly proportional to the level and duration of oxidative stress, which is the primary cause. During this process, inflammatory cell infiltration in the pulmonary tissue induces the release of reactive oxygen metabolites, as well as cytokines and proteolytic-lipolytic enzymes from these cells, after which these mediators of oxidative stress increase alveolocapillary membrane permeability and microvascular leakage associated with the formation of intra-alveolar hemorrhage and alveolar edema fluid. We found that Erdosteine yielded the expected potent antioxidant effect and that the level of pulmonary tissue damage was reduced, which in turn led to a decrease in the level of intra-alveolar hemorrhage. We also determined that congestion and leukocyte infiltration were significantly decreased in Group B. Oxidative stress and the accompanying severe inflammation resulted in vasodilatation and dense congestion as a secondary effect. Erdosteine inhibited the migration of inflammatory cells in Group B to the area of tissue damage, therefore, suppressing the inflammation and reducing the severity of congestion. Leukocyte infiltrations were typically observed 6 to 24 hours after such operations. Although the rats in our study were sacrificed within 3 hours of administration of CO_2 _pneumoperitoneum and the conclusion of the trial, severe leukocyte infiltration was detected in the control group. We attribute this finding to the relatively higher pressure of 15 mmHg used in CO_2 _pneumoperitoneum. The duration, however, is less important than pressure with regards to hemodynamic effects and complications that may potentially develop with pneumoperitoneum. The use of a low-pressure pneumoperitoneum may reduce the hazardous effects of ischemia/insufflation and reperfusion/deflation periods. Gutt et al. suggested that intra-abdominal pressure maintained at moderate to low levels (<12 mmHg) while administering CO_2 _pneumoperitoneum can help limit the extent of the pathophysiological changes and minimize or make transient any potential organ dysfunction and complications [4]. Our findings are consistent with those of other studies. For example, in a study of the effects of Erdosteine on acute inflammatory changes and fibrosis, Erden et al. concluded that the drug inhibits acute inflammation by preventing the migration of neutrophils to the inflammation site and blocking lipid peroxidation. They noted that the protective effect of Erdosteine was due to its removal of free radicals from the environment and its antioxidant activity [18]. Moretti et al. reviewed acute injury induced by a variety of pharmacological or noxious agents. They concluded that Erdosteine prevents the accumulation of free oxygen radicals when their production is accelerated and increases antioxidant cellular protective mechanisms, thereby reducing lipid peroxidation, neutrophil infiltration, or cell apoptosis mediated by noxious agents [16].

Although the causes of alveolar edema include tissue inflammation and congestion, we did not detect any significant alveolar edema in either group during our study. We are unable to explain fully the pathophysiological and histological basis of this result. However, alveolar edema is a dynamic phenomenon, and its development is associated with the disturbance of the balance between the mechanisms that force the formation and increase the clearance of the phenomenon [22]. Therefore, one potential explanation may be that the mechanisms running in contrast with each other during the trial were all in balance.

Limitations of this experimental study include the low number of rats, the short postoperative time, and the lack of use of various doses of Erdosteine. Our findings are also based on the result of histopathological examination. Biochemical data would elucidate physiopathological changes associated with CO_2 _pneumoperitoneum-induced oxidative damage and the effects of Erdosteine. Experiments involving a higher number of rats and a longer postoperative duration may yield more comprehensive results. The value of the data obtained in this study will benefit from future studies that include different doses of Erdosteine and time protocols and possibly different application methods and that biochemically determine free oxygen radicals, antioxidant enzymes, and lipid peroxidation products in tissue and blood.

## Conclusion

The present study demonstrates that CO_2 _pneumoperitoneum results in oxidative stress injury to lung tissue and that the prophylactic administration of Erdosteine could reduce the severity of pathological changes in the lungs. Thus, Erdosteine seems to be a useful preventive and therapeutic agent for CO_2 _pneumoperitoneum-induced oxidative stress and inflammatory reactions. Although these findings are not transferrable to clinical practice, they highlight the future potential of this treatment protocol in managing pulmonary complications with CO_2 _pneumoperitoneum in laparoscopic surgery. Ultimately, the potential will depend on the results of clinical Phase 1 and Phase 2 studies of Erdosteine administered to human subjects.

## Competing interests

The authors declare that they have no competing interests.

## Authors' contributions

SK, SG, TD and BK participated in the design of the study and coordination, literature search, data analysis, and writing/revision of manuscript. UY carried out the analysis of the pathological sections. IO contributed to the surgical procedure. MY helped with surgical techniques. AI, HK and AS supervised the study and performed the statistical analysis. All authors read and approved the final manuscript.
